# Optimizing real-time phase detection in diverse rhythmic biological signals for phase-specific neurostimulation

**DOI:** 10.1088/1741-2552/ae10e1

**Published:** 2025-10-24

**Authors:** Mengzhan Liufu, Zachary M Leveroni, Sameera Shridhar, Nan Zhou, Jai Y Yu

**Affiliations:** 1 Department of Computer Science, University of Chicago, Chicago, IL 60637, United States of America; 2 Department of Psychology, University of Chicago, Chicago, IL 60637, United States of America; 3 Institute for Mind and Biology, University of Chicago, Chicago, IL 60637, United States of America; 4 Neuroscience Institute, University of Chicago, Chicago, IL 60637, United States of America

**Keywords:** phase detection, closed-loop, real time, neurostimulation, algorithm, rodent, human

## Abstract

*Objective.* Closed-loop, phase-specific neurostimulation is a powerful method to modulate ongoing brain activity for clinical and research applications. Phase-specific stimulation relies on estimating the phase of an ongoing oscillation in real time and issuing a control command at a target phase. Phase detection algorithms based on the fast Fourier transform (FFT) are widely used due to their computational efficiency and robustness. However, it is unclear how algorithm performance depends on the spectral properties of the input signal and how algorithm parameters can be optimized. *Approach.* We evaluated the *in silico* performance of three phase detection algorithms [Endpoint-corrected Hilbert transform (ecHT), Hilbert transform (HT), and phase mapping (PM)] on three real-world biological signals with distinct spectral properties (theta oscillations from rodent hippocampal local field potential, alpha oscillations from human electroencephalogram (EEG), and hand movement kinematics from essential tremor patients) to identify the optimal model and parameters. We then validated the performance of an algorithm for estimating theta phase in real-time using rats implanted with electrodes in the hippocampus. *Results*. First, we found that signal amplitude and frequency variations strongly influence algorithm performance. Frequency-specific signal-to-noise ratio was positively correlated with performance (mean *R*^2^ = 0.42 across metrics), while amplitude and frequency variability were negatively correlated (mean *R*^2^ = 0.50 across metrics). Second, we showed that the length of the data window used for phase estimation is the key parameter for optimal performance of FFT-based algorithms, where the optimal data window length corresponds to the period of the oscillation (∼150 ms for hippocampal theta oscillations, ∼100 ms for human EEG alpha, and ∼125 ms for essential tremor kinematics). We validated this finding *in vivo* by estimating the phase of theta oscillations from the hippocampus of freely behaving rats, where a data window length corresponding to one theta cycle yielded the best performance across all metrics compared with shorter or longer window lengths. *Significance*. Our findings clarify the relationship between signal properties and algorithm performance and provide a convenient method for optimizing FFT-based phase detection algorithms. We show that a data window length corresponding to one cycle of an oscillation can lead to improved performance.

## 1. Introduction

Closed-loop, phase-specific neurostimulation is an approach to modulate the nervous system contingent on information from oscillating biological signals detected from the body. This technique has been effectively used in clinical settings to manage a range of neurological disorders and in basic research to investigate the contribution of neural oscillations to brain function (Widge and Miller 2019, Wendt *et al* 2022, Widge 2024). Phase-specific stimulation systems rely on phase detection algorithms that (1) estimate the phase of an ongoing oscillating signal and (2) issue outputs once a specific phase target has been detected. A commonly used approach for phase estimation first applies fast Fourier transform (FFT)-based bandpass filtering to identify and isolate the frequency band of interest, and then uses the Hilbert transform (HT) to compute instantaneous phase. Simple FFT with HT methods have the advantage of convenient implementation, well-characterized performance and wide adoption in the community (Rosenblum *et al* 2021, Wodeyar *et al* 2021). Standard signal processing libraries for these methods are available in many coding languages, which supports tuning of parameters for each application.

Reliable phase detection is essential for real-time applications, but the optimization of algorithm performance faces several challenges since performance depends on multiple algorithm parameters and the interaction between the algorithm and the input signal. First, it remains unclear how the spectral properties of the input signal affect performance. Prior work suggests that signal amplitude and signal-to-noise ratio (SNR) are major determinants of algorithm performance (Mansouri *et al* 2017, Shirinpour *et al* 2020, Kim *et al* 2023). However, other work points to temporal properties, such as signal frequency and amplitude variability, being correlated with performance (Mansouri *et al* 2017). It remains unclear how these signal properties can be used to guide the optimization of algorithm performance. Second, it is unclear how algorithm performance can be optimized by systematically adjusting algorithm parameters. While signal properties affect the choice of parameters for optimal algorithm performance, the relationship between signal properties and optimal algorithm parameters is non-trivial. Identifying the optimal parameters required searching through many possible combinations (Chen *et al* 2013).

Our study addresses these challenges. We evaluate the performance of phase detection algorithms in two use cases: generating outputs at a desired target phase consistently over time, or at random phases on each cycle. We evaluate the algorithm output with four metrics that quantify how closely the algorithm outputs match the desired phase target and the rhythmicity of the output sequences in time. These algorithms are applied to three rhythmic biological signals with diverse spectral properties, recorded in real-world laboratory and clinical settings. We determine the relationship between signal properties and algorithm performance. Lastly, we identify optimal algorithm parameters and validate our predictions with a real-time *in vivo* experiment.

## 2. Results

### 2.1. Metrics for evaluating algorithm output

**Figure 1.**
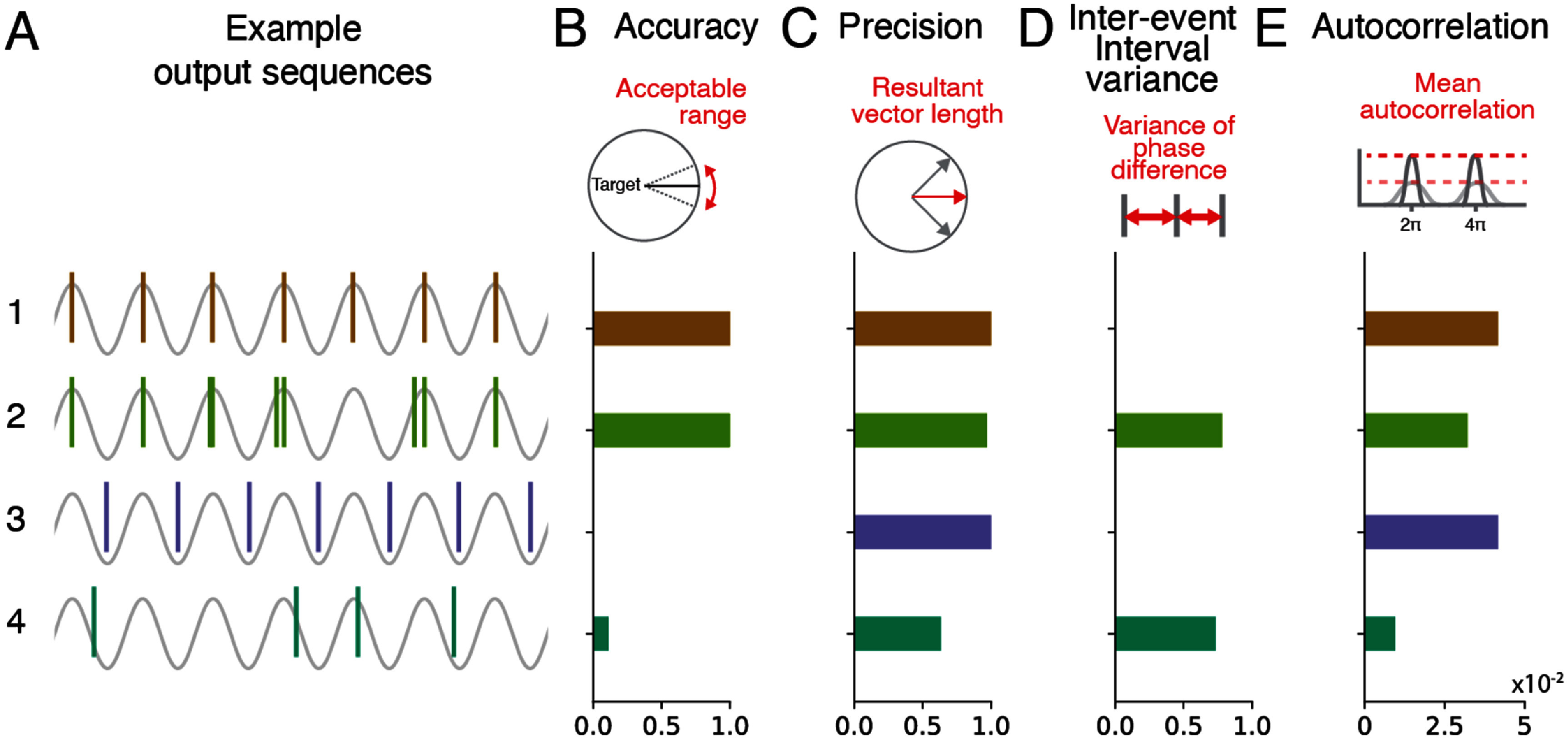
Metrics for evaluating the performance of phase detection algorithms. (A) Example output event sequences from a phase detection algorithm where the target for detection is at the peak of each cycle. This illustrates several scenarios with distinct output properties. The underlying sine signal is shown in gray. Hypothetical algorithm output is shown as vertical lines. Each color represents a distinct scenario. 1: The algorithm generated consistent output events at peaks of the rhythm. 2: The algorithm generated output events within a *π*/4 range centered around the peak but skipped events on 20% of the cycles and repeated events in 30% of the cycles. 3: The algorithm generated consistent output events at the trough despite the target being the peak. 4: The algorithm generated random events. This was generated from a von Mises distribution (*μ* = *π, κ* = 0). 10% of the cycles were skipped. (B) Algorithm output accuracy in each scenario quantified by the proportion of events within a *π*/4 window centered around the target phase (peak). (C) Algorithm output precision in each scenario quantified by the resultant vector length of event phases. (D) IEI variance in each scenario quantified by the variance of phase differences between events. (E) Autocorrelation in each scenario quantified by the mean autocorrelation values at integer multiples of 2*π* over an 80-radian window. In the schematic, the solid red dotted line and solid gray autocorrelation curve represent the autocorrelation of regular algorithm output phases with high autocorrelation values at multiples of 2*π*. The semi-transparent red dotted line and gray curve represent the autocorrelation of less regular algorithm output phases.

When phase detection algorithms are used for phase-specific stimulation, stimulation triggers are issued at a specific phase within each cycle of the oscillation. The goal of a phase-specific stimulation system is to produce a sequence of output events that reliably match the desired phase target of the underlying oscillation. The system should consistently produce an output in each cycle, avoiding skipped cycles or repeated outputs within the same cycle (figure 1(A)). The resulting output triggers should be a rhythmic sequence in time and at the desired target phase.

Given these goals, we identified four metrics that capture different properties of the output sequence. Two widely used metrics are accuracy and precision. Accuracy reports the proportion of output events within an acceptable range relative to the selected target phase (figure 1(B)). Precision reports the consistency of output events and is quantified using the resultant vector length (figure 1(C)). In addition to quantifying whether outputs are issued at the correct target phase, we chose two metrics to quantify the rhythmicity of the output sequence in time: inter-event interval (IEI) variance and autocorrelation. The IEI is the phase difference between two consecutive output events (figure 1(D)). In an ideal case, where each event is delivered consistently at the phase target, the output sequence will only have IEIs of 2*π*. We calculate the variance of the IEIs as a measure of consistency in time. This metric will detect deviations such as multiple output events per cycle or missed events over consecutive cycles. To assess the rhythmic regularity of the output events across cycles, we calculated autocorrelation for the algorithm output sequence, where each value corresponds to a phase value (see Methods). If the algorithm consistently outputs at the same phase of each oscillatory cycle, the intervals between detections should align with multiples of 2*π*. In such cases, the autocorrelation of the detection phases shows clear peaks at those intervals, reflecting strong rhythmic regularity (figure 1(E)). If the algorithm produces outputs that occur at irregular phases, the autocorrelation will have weaker periodic peaks. We calculated the mean autocorrelation values at integer multiples of 2*π*. Higher values indicate more consistent algorithm outputs, while lower values suggest less regular output.

To illustrate how these different metrics can be used to evaluate the outputs generated from an algorithm, we provide four example output sequences that vary in phase consistency or rhythmicity. Example sequence 1 is an ideal output for detecting the peak of an oscillation. All the output events are perfectly aligned to the peak, which leads to maximum accuracy, precision and autocorrelation, and zero IEI variance. Example sequence 2 has extra, undesired events close to the target phase in some cycles and has missing events in other cycles. In this case, the phase accuracy and precision remain similar to sequence 1 but IEI variance and autocorrelation reveal decreased rhythmicity. Example sequence 3 is perfectly consistent and rhythmic in time, but all the output events occur at the trough of the cycle, which is not the target. This sequence scores high in the rhythmicity metrics but low in accuracy. Example sequence 4 was generated from a random distribution; the sequence is neither consistent in phase nor rhythmically regular.

### 2.2. Evaluating algorithm performance on rhythmic biological signals

**Figure 2.**
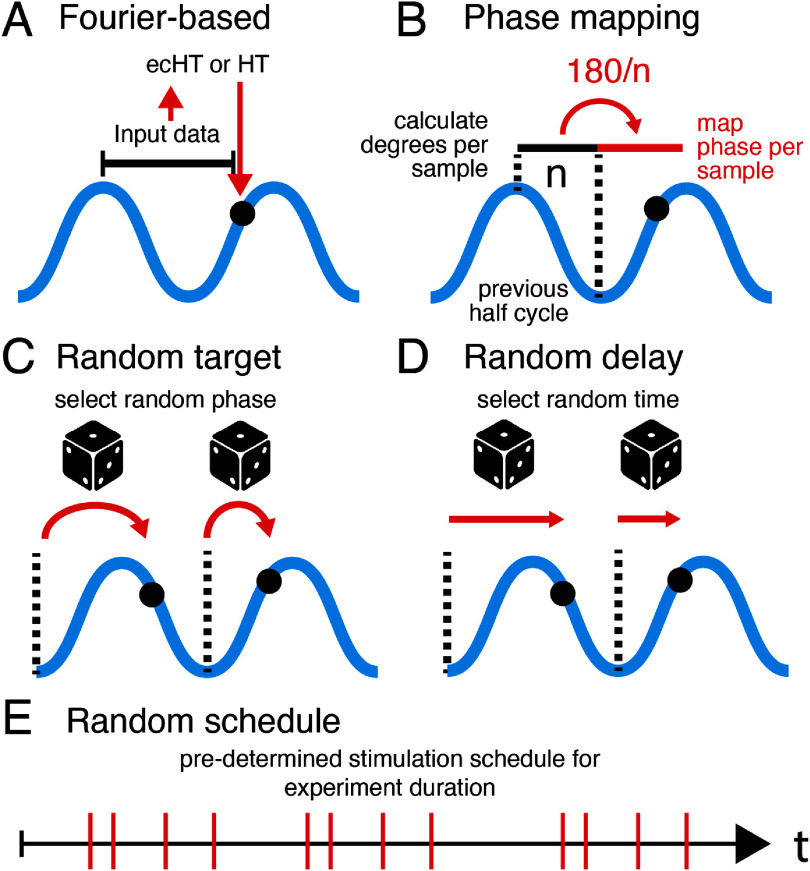
Phase detection and random phase generating algorithms. (A) Schematic of the Fourier-based ecHT and HT algorithms. The filtered signal is shown in blue. The input data window used for estimating phase is shown as the horizontal black line. The algorithm output is the black circle. (B) Schematic of the PM algorithm. The window used to calculate the degrees per sample is the horizontal black line. This is used to map the degrees per sample in the next half-cycle shown in red. The algorithm output is the black circle. (C) Schematic of the random target algorithm. The vertical dotted lines represent the trough of each cycle, where the algorithm resets for each cycle. At this point, a random phase target is selected (dice symbol) and the algorithm produces an output when the target is reached (curved red arrow) on that cycle (black circle). (D) Schematic of the random delay algorithm. The vertical dotted lines represent the trough of each cycle, where the algorithm resets for each cycle. At this point, a random time is selected (dice symbol) and the algorithm produces an output when the time is reached (horizontal red arrow) on that cycle (black circle). The random time generator should draw from a distribution with a mean that matches the average cycle duration for the frequency of interest. (E) Schematic of the random schedule algorithm. The output events (red vertical lines) are generated according to a set schedule that was predetermined based on the duration of the experiment (horizontal line with arrow).

**Figure 3.**
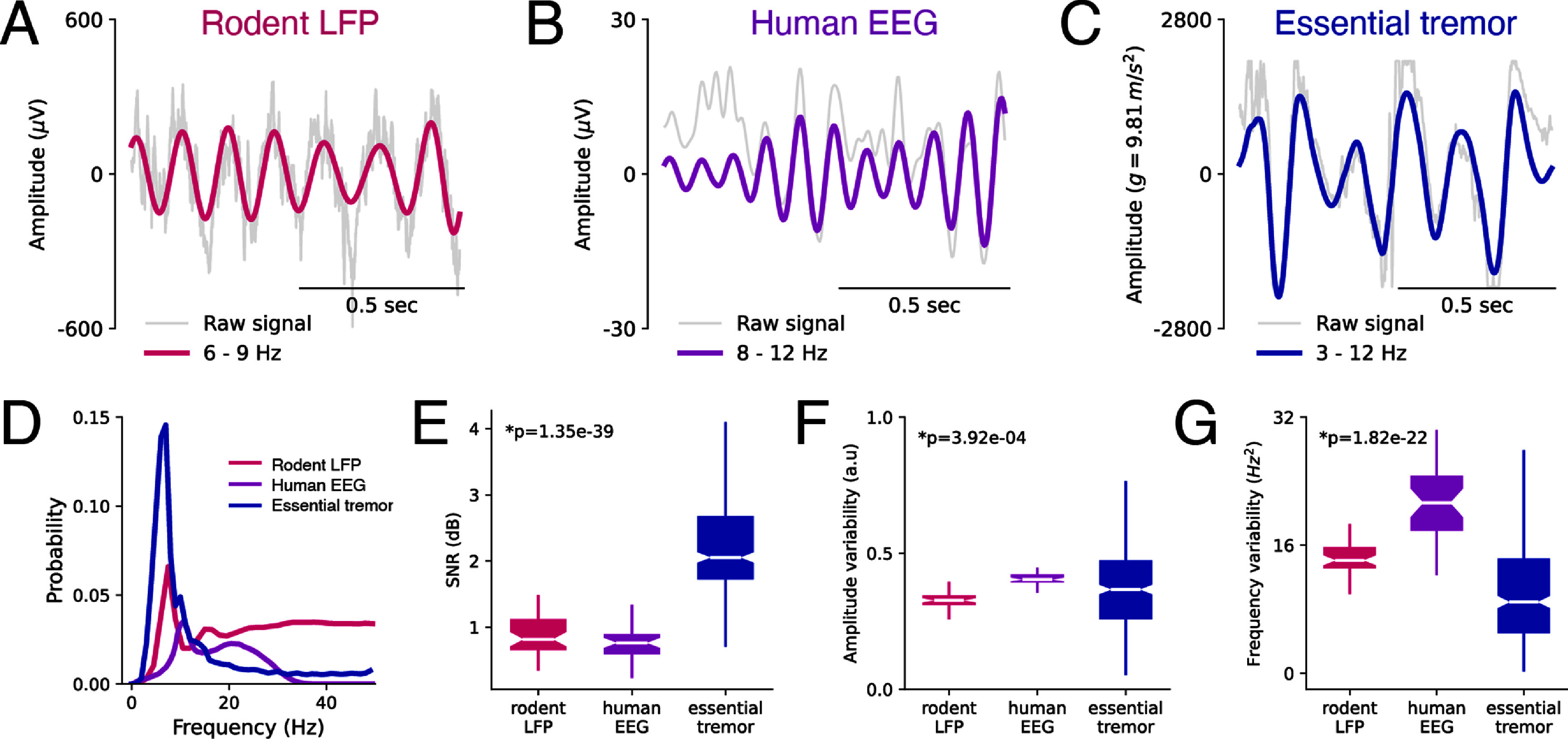
Spectral properties for three rhythmic biological signals. (A) Example rodent LFP from the dorsal CA1 of the hippocampus (gray), filtered in the theta range (6–9 Hz) (magenta). (B) Example human EEG from the POZ electrode (gray), filtered in the alpha range (8–12 Hz) (purple). (C) Example hand acceleration trace from human essential tremor patients (gray), filtered in the tremor range (3–12 Hz) (blue). (D) Mean spectrograms of sample signals. Rodent LFP (pink, *n* = 11, duration = 40 min each). Human EEG (purple, *n* = 40, duration = 50 min each). Human essential tremor (blue, *n* = 11, duration = 30 min each). (E) Box plot of SNR distributions. Statistical differences across signal type were tested using the Kruskal–Wallis test (*h* = 1.96 × 10^2^, *p* = 3.11 × 10^−43^). (F) Box plot of amplitude variability distributions. Statistical differences across signal type were tested using the Kruskal–Wallis test (*h* = 1.57 × 10^1^, *p* = 3.92 × 10^−4^). (G) Box plot of frequency variability distributions. Statistical differences across signal type were tested using the Kruskal–Wallis test (*h* = 1.00 × 10^2^, *p* = 1.82 × 10^−22^).

We next used these metrics to evaluate the performance of three phase detection algorithms: the Hilbert transform (HT) algorithm (Chen *et al* 2013, Blackwood *et al* 2018, Knudsen and Wallis 2020, Shirinpour *et al* 2020, Zrenner *et al* 2020); the endpoint-corrected HT (ecHT) algorithm (Schreglmann *et al* 2021, Bressler *et al* 2024) (figure 2(A)) and the phase mapping (PM) algorithm (figure 2(B)). To understand how these algorithms perform on different rhythmic biological signals and how performance depends on signal properties, we selected three real-world biological signal datasets with diverse spectral properties: rodent local field potential (LFP) from the dorsal CA1 region of the hippocampus (figure 3(A)), human electroencephalogram (EEG) from the POZ electrode over the visual regions in posterior brain (Unsworth *et al* 2014) (figure 3(B)) and hand acceleration kinematics of essential tremor patients measured from an accelerometer attached to the middle finger of their dominant hand (Brittain *et al* 2013, Schreglmann *et al* 2021) (figure 3(C)). For each signal, we extracted the oscillations in the frequency band with known physiological correlates, tremor frequency for hand kinematics (3–12 Hz) (Brittain *et al* 2013, Cagnan *et al* 2013, Louis and Faust 2020, Schreglmann *et al* 2021, Welton *et al* 2021); alpha oscillation for human EEG (8–12 Hz) (Klimesch *et al* 2003, Halgren *et al* 2019, Peylo *et al* 2021, kim *et al* 2023, Pantazatos *et al* 2023, Bressler *et al* 2024); and theta oscillation for rat LFP (6–9 Hz) (Buzsáki 2002, Hyman *et al* 2003, Buzsáki and Moser 2013, Siegle and Wilson 2014, Lurie *et al* 2022) (figure 3(D)). These oscillations differ in SNR_freq. band_ (Kruskal–Wallis test, H (2, 424) = 196, *p* < 1 × 10^−42^); amplitude variability over time as measured by normalized pairwise variability index (nPVI) (Kruskal–Wallis test, H (2, 424) = 15.7, *p* < 1 × 10^−21^); and frequency variability (Kruskal–Wallis test, H (2, 424) = 101, *p* < 1 × 10^−3^) (figures 3(E)–(G)). We define SNR_freq. band_ as the ratio of the power within the frequency band of interest to aperiodic power within that band (see Methods) (Zrenner *et al* 2020). This measures the relative strength of the oscillatory component versus the general background power in the signal for the frequency band of interest. Since SNR has different definitions, we note that SNR_freq. band_ used for our analyses is different from electrical or motion artifacts in the signal, and it is different from the strength of the oscillation in the frequency band of interest relative to the power across all frequencies (Shirinpour *et al* 2020).

Next, we sought to identify which algorithm parameters can be optimized to improve performance. We identified three main parameters for these algorithms that can be tuned for optimization: the type of bandpass filter, the order of the filter, and the input data window length (the number of data points in the past used for estimating the current phase) (Chen *et al* 2013). We tuned the parameters for each algorithm using a grid search. Specifically, we ran each algorithm using combinations of parameter values that spanned the parameter space and analyzed how each parameter affected performance. This was done to detect cycle peaks in real data from all three signal types. Our optimization space spans three commonly used bandpass filters (Butterworth, Chebyshev type I, Elliptic), filter orders from 2 to 6, and input data window length ranging from 50 ms to 500 ms. We then quantified algorithm output from each parameter combination using our four performance metrics and analyzed how each parameter affects performance. First, we found that filter type does not significantly affect algorithm performance (Supp. figure 1, Supp. table 1–4). There was only a significant difference between filter types for PM in rodent LFP, with the Butterworth filter outperforming the other two. We selected the Butterworth filter for further optimization because of its even frequency response compared with the other two filter types. We next examined the effect of filter order on algorithm performance. Overall, filter order correlates negatively with algorithm performance (Supp. figures 2, 3). The accuracy and precision of algorithm output are more sensitive to filter order than IEI variance and autocorrelation. We therefore continued optimization with a filter order 2. For PM, any filter order above 2 is not only suboptimal but unviable, because higher filter order drastically reduces the power of the signal derivative required by PM to detect the peak and trough within a cycle (Supp. figures 4, figure 2(B)).

**Figure 4.**
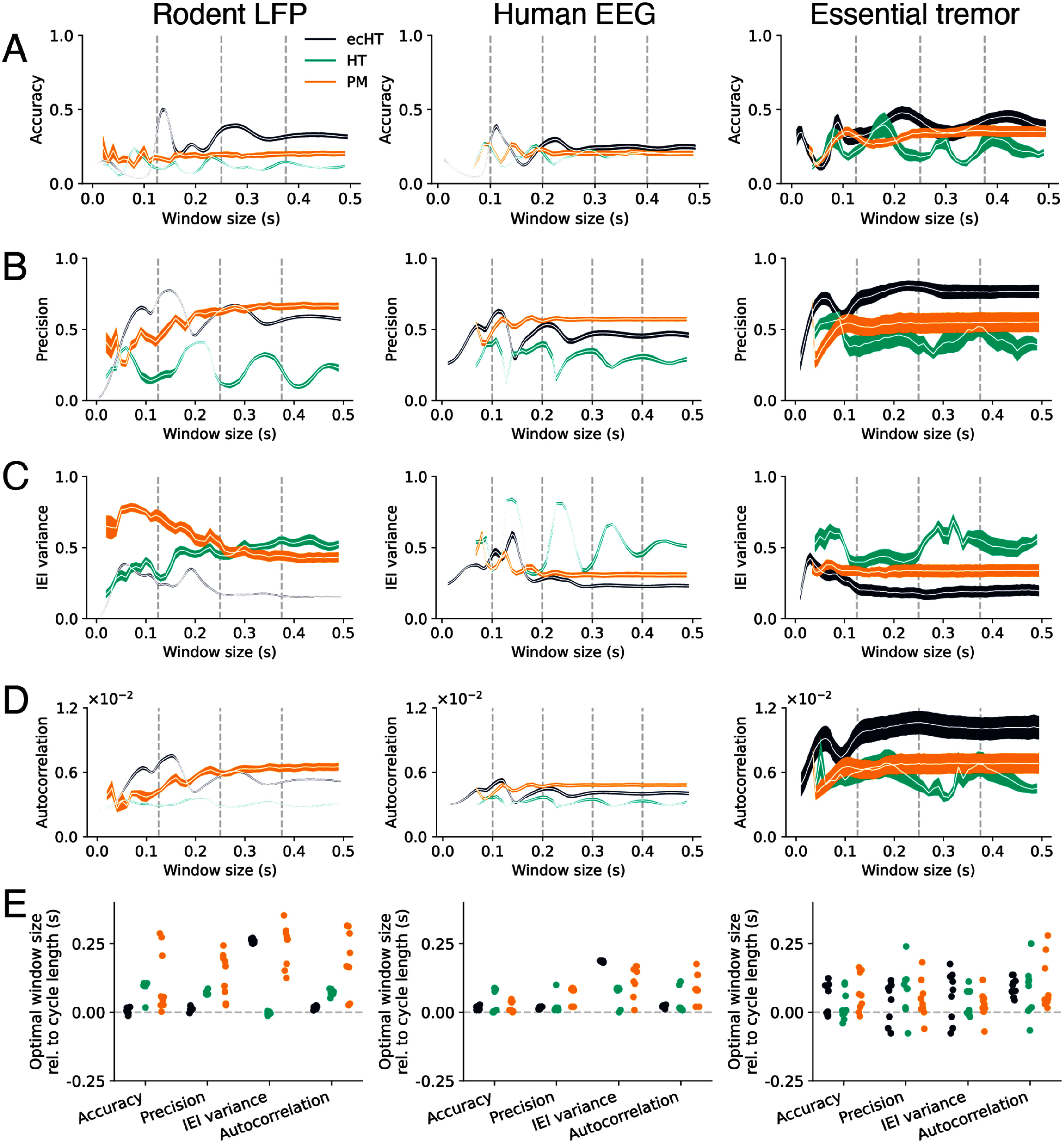
Relationship between algorithm performance and input data window length. (A) Accuracy of algorithm output varies with input data window length (ecHT: black, HT: green, and PM: yellow) for rodent LFP (left), human EEG (center) and essential tremor (right) data. All algorithms used a Butterworth filter at filter order 2. Mean and standard error are shown for each algorithm. Vertical dotted lines mark window lengths that correspond to multiples of the oscillation cycle length for each signal (rodent LFP: 125 ms, human EEG: 100 ms, and essential tremor: 125 ms). (B) Precision of algorithm output varies with input data window length. (C) IEI variance of algorithm output varies with input data window length. (D) Autocorrelation of algorithm output varies with input data window length. (E) Difference between optimal window length and oscillation cycle length for each performance metric, algorithm (ecHT: black, HT: green, and PM: yellow) and signal type (rodent LFP: left, human EEG: center. and essential tremor: right). Each point represents data from one individual.

We then examined the effect of input data window length and found this to be an important factor in algorithm performance. The optimal window length corresponds approximately to the length of one cycle of the oscillation. The performance of ecHT and HT varied significantly across a range of window lengths and differed between signal types (figures 4(A)–(D)). The performance of PM was less sensitive to window length, since it does not require a time-frequency transformation. The window length yielding the best accuracy, precision, and autocorrelation was close to the cycle length for each frequency of interest (figure 4(E)). The optimal window length for reduced IEI variance was slightly above twice the cycle length. Further, we observed periodicity in the performance metrics, where local maxima occurred at multiples of the cycle length. We found differences across signals in how well cycle length correlates with the optimal window length. The window length for optimal algorithm performance is closest to the cycle length for rodent LFP and human EEG signals, compared with human essential tremor signals. These results point to a trade-off between phase reliability and temporal consistency in the algorithm output. An input data window containing one cycle of signal produces optimal accuracy, precision and autocorrelation, but slightly longer windows minimize IEI variance. An input window containing more than two cycles of signal did not produce performance benefits.

The effect of window length on algorithm performance can be explained by the amount of frequency variability within the window. Previous work found that frequency variability of the entire recording is negatively correlated with algorithm performance (Shirinpour *et al* 2020). We show that frequency variability on a shorter timescale is critical for algorithm performance as well. Specifically, we found that frequency variability within a window increases with window length and approaches the variability of the entire recording as the length of the window increases (Supp. figure 5). This was determined by calculating the frequency variability within data windows of different lengths, expressed as a fraction of the frequency variability for the entire signal (see methods). Although previous work did not specify how frequency variability may worsen performance, we hypothesize that increased frequency variability within the window can worsen phase estimation performance. We confirmed this hypothesis by running the algorithms with window length corresponding to the duration of one cycle using recorded data. We correlated frequency variability for this window length with the resulting phase estimation error and found consistently positive correlation for all algorithms and signal types (Supp. figures 4(D)–(F)). Thus, longer data window leads to larger phase estimation errors because of the increased frequency variability. This suggests that optimal performance can be achieved by setting window length to control frequency variability.

**Figure 5.**
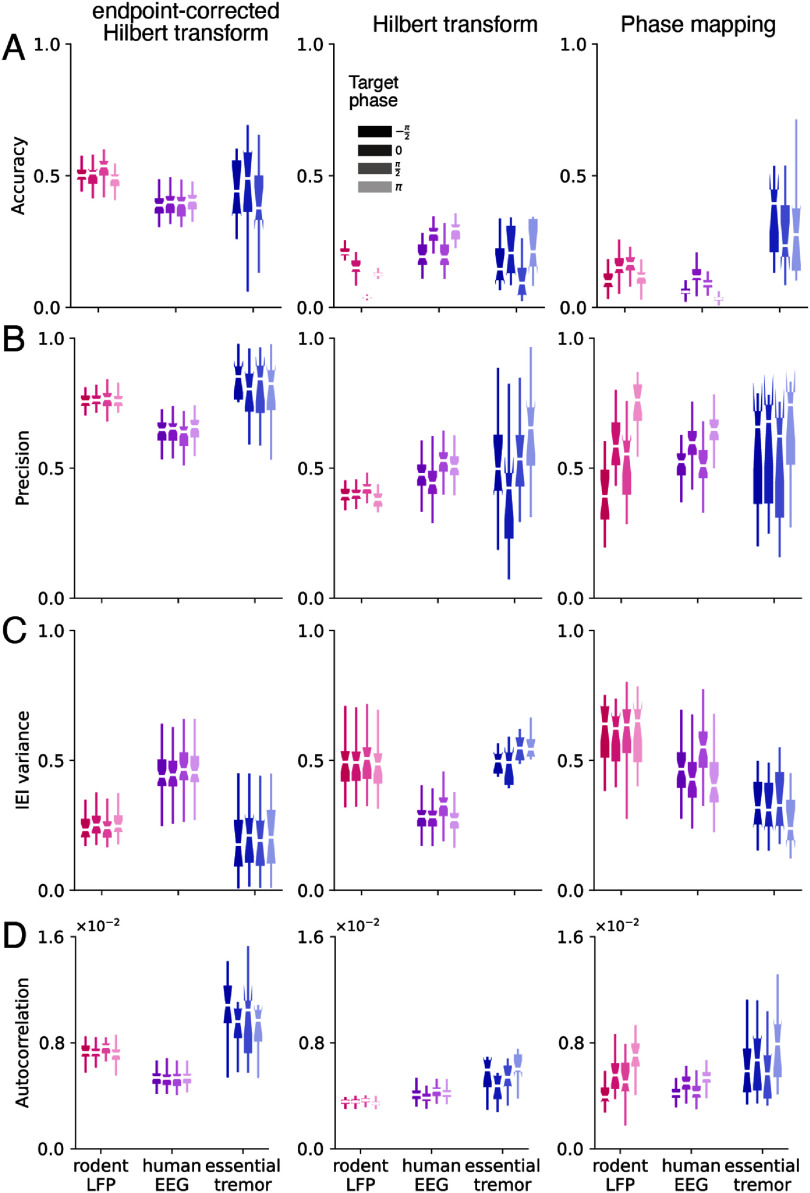
Algorithm performance depends on signal type. (A) Accuracy of algorithm output varies between signal types (rodent LFP: pink, human EEG: purple, essential tremor: blue) and algorithm (ecHT: left, HT: center, PM: right). The input data window has been fixed at the optimal size based on results from figure 4. Each algorithm was run to detect four target phases (−*π*/2, 0, *π*/2, *π*). Shading indicates the target phase (from darkest to lightest: −*π*/2, 0, *π*/2, *π)*. (B) Precision of algorithm output varies between signal types. (C) IEI variance of algorithm output varies between signal types. (D) Autocorrelation of algorithm output varies between signal types.

Having determined the parameters for each algorithm, we then compared their performance across signals. We applied the algorithms to each type of real-world data to detect four evenly distributed phase targets across the cycle, −*π*/2 (rising), 0 (peak), *π*/2 (falling), *π* (trough) (figure 5). For each algorithm, performance differed across signal types (table 1, *p*-value of signal type < 0.05 for all metrics). Rodent LFP and human EEG data yielded the most consistent outcomes across individual subjects for all algorithms (Levene’s test, F (2, 424) > 10, *p* < 1 × 10^−5^ for all metrics. *p* < 1 × 10^−2^ for all post-hoc pairwise comparisons). There was an overall performance difference between the algorithms (table 1, *p*-value of algorithm < 5 × 10^−2^ for all metrics), with ecHT yielding better performance compared with PM or HT across all metrics (Tukey’s HSD test, *p* < 1 × 10^−2^ for all pairwise comparisons). Algorithm performance varied across the four phase targets, with ecHT being the most consistent across target phases. Additionally, we found that ecHT had the best on-target performance, as shown by the distributions of algorithm output compared with the four corresponding phase targets (Supp. figure 10). ecHT consistently had the least bias (<0.2 radians), compared with much larger biases for the other algorithms (Supp. table 6).

**Table 1.** Three-way permutation-based ANOVA of the effects of signal type, algorithm and target phase on each algorithm performance metric.

Performance metric	Source of variation	df	SS	MS	F	p-value
Accuracy	Target phase	3	0.7356	0.2452	30.3902	1.33 × 10^−18^
	Algorithm	2	19.9466	9.9733	1236.1771	2.70 × 10^−244^
	Signal type	2	1.5098	0.7549	93.5673	3.32 × 10^−37^
Precision	Target phase	3	0.2583	0.0861	9.7180	2.65 × 10^−06^
	Algorithm	2	8.3663	4.1831	472.2083	9.82 × 10^−136^
	Signal type	2	0.8372	0.4186	47.2504	4.11 × 10^−20^
IEI variance	Target phase	3	0.3197	0.1066	14.6642	2.66 × 10^−09^
	Algorithm	2	0.2262	0.1131	15.5641	2.34 × 10^−07^
	Signal type	2	0.3100	0.1550	21.3322	9.47 × 10^−10^
Autocorrelation	Target phase	3	1.7 × 10^−06^	6.0 × 10^−07^	3.0279	2.87 × 10^−02^
	Algorithm	2	8.64 × 10^−05^	4.32 × 10^−05^	234.2373	1.21 × 10^−80^
	Signal type	2	1.02 × 10^−04^	5.11 × 10^−05^	276.8501	6.78 × 10^−92^

**Figure 6.**
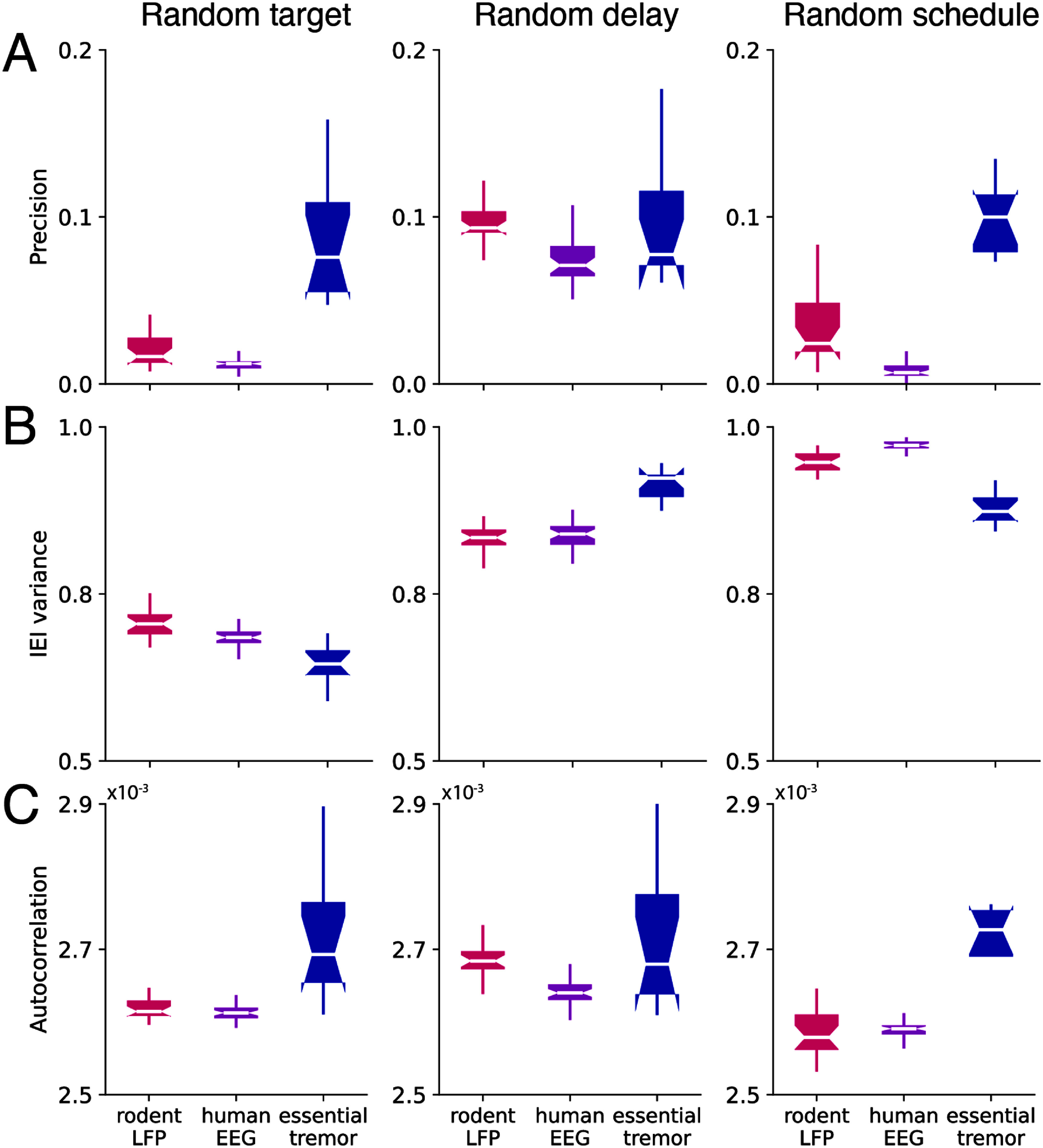
Performance of random-phase-generating algorithm depends on signal type. (A) Precision of random-phase_generating algorithm output varies between signal types (rodent LFP: pink, human EEG: purple, essential tremor: blue) and random-phase-generating algorithm (random target: left, random delay: center, random schedule: right). (B) IEI variance of random-phase_generating algorithm output varies between signal types. (C) Autocorrelation of random-phase_generating algorithm output varies between signal types.

**Figure 7.**
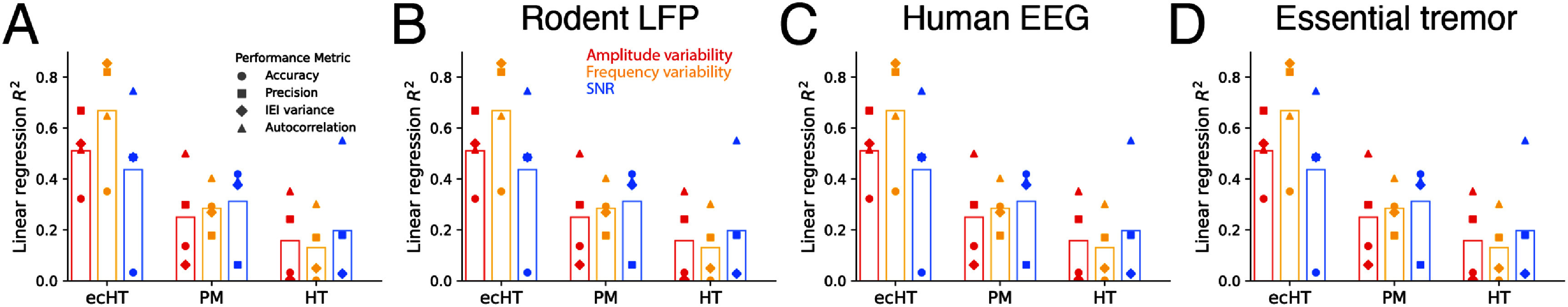
Signal amplitude and frequency variability affect algorithm performance. (A) *R*^2^ value of linear correlation between each signal property and each algorithm performance metric across signal types. Color indicates signal property (red: amplitude variability, yellow: frequency variability, blue: frequency-specific SNR). Shape indicates performance metric (circle: Accuracy, square: Precision, diamond: IEI variance, triangle: Autocorrelation). The mean *R*^2^ scores are indicated by the vertical bars. (B) *R*^2^ value of linear correlation between signal property and algorithm performance metric for rodent LFP. (C) *R*^2^ value of linear correlation between signal property and algorithm performance metric for human EEG. (D) *R*^2^ values of linear correlations between signal property and algorithm performance metric for essential tremor.

We next analyzed the performance of algorithms that generate outputs without phase specificity, which we refer to as random-phase-generating algorithms. Laboratory experiments using phase-specific stimulation often require a non-phase-specific stimulation condition as control. The control helps establish that the observed effects are specific to a unique stimulation phase for the rhythmic signal of interest (Ngo *et al* 2013, Hampson *et al* 2018, Zanos *et al* 2018, Kanta *et al* 2019, Knudsen and Wallis 2020, West *et al* 2022, Dong *et al* 2023, Xie *et al* 2023, Roeder *et al* 2024). We evaluated the output of three random-phase-generating algorithms (random target, random delay and random schedule) (figures 2(C)–(E), figure 7). There was an overall difference between their outputs (table 2, *p*-value of algorithm < 0.05 for all metrics). The random target algorithm produced the most random output phases as measured by resultant vector length (Tukey’s HSD test, *p* < 0.05 for all pairwise comparisons) (figure 6(A)). The random schedule and random target algorithms produced the most arrhythmic sequences as measured by IEI variance and autocorrelation (Tukey’s HSD test, *p* < 0.05 for all pairwise comparisons) (figures 6(B) and (C)). The algorithm performance was again dependent on signal type (table 2, *p*-value of signal type < 0.05 for all metrics) with rodent LFP and human EEG yielding the most random and arrhythmic outcomes. Essential tremor signals yielded less random output overall (Tukey’s HSD test, *p* < 0.05 for all pairwise comparisons) and less consistent autocorrelation across individuals (Levene’s test, F (2, 424) = 21.08, *p* = 6.73 × 10^−09^. *p* < 0.05 for all post-hoc pairwise comparisons).

**Table 2.** Two-way permutation-based ANOVA of the effects of signal type and algorithm on each algorithm performance metric.

Performance metric	Source of variation	df	SS	MS	F	p-value
Precision	Algorithm	2	0.1447	0.0724	150.4087	1.88 × 10^−40^
	Signal type	2	0.1124	0.0562	116.8723	3.04 × 10^−34^
IEI variance	Algorithm	2	2.3482	1.1741	2062.7140	2.65 × 10^−133^
	Signal type	2	0.0097	0.0049	8.5439	2.76 × 10^−04^
Autocorrelation	Algorithm	2	1.92 × 10^−07^	5.46 × 10^−08^	14.4489	1.40 × 10^−06^
	Signal type	2	3.16 × 10^−07^	1.58 × 10^−07^	41.7439	7.90 × 10^−16^

### 2.3. Signal properties affect algorithm performance

We next asked how the spectral properties of a signal are correlated with algorithm performance. We performed linear regression between each signal property and each algorithm performance metric (Supp. figures 6–8). For all signal types and phase-specific algorithms, frequency-specific SNR was positively correlated with algorithm performance, and amplitude and frequency variability were negatively correlated with performance. For all signal types, the performance variations of ecHT consistently showed strong correlations with amplitude and frequency variability. For PM and HT, signal properties were less correlated with algorithm performance (figure 7). We compared the explained variance (*R*^2^) of linear regressions between signal properties. We found that frequency-specific SNR is correlated with algorithm performance, which is consistent with previous findings (Shirinpour *et al* 2020, Zrenner *et al* 2020, Rodriguez Rivero and Ditterich 2021, Wodeyar *et al* 2021, Kim *et al* 2023). In addition, we found that signal amplitude and frequency variability are also significantly correlated with algorithm performance. That is consistent with our earlier finding that increased frequency variability is correlated with increased phase estimation error.

### 2.4. *In vivo* validation of algorithm optimization

**Figure 8.**
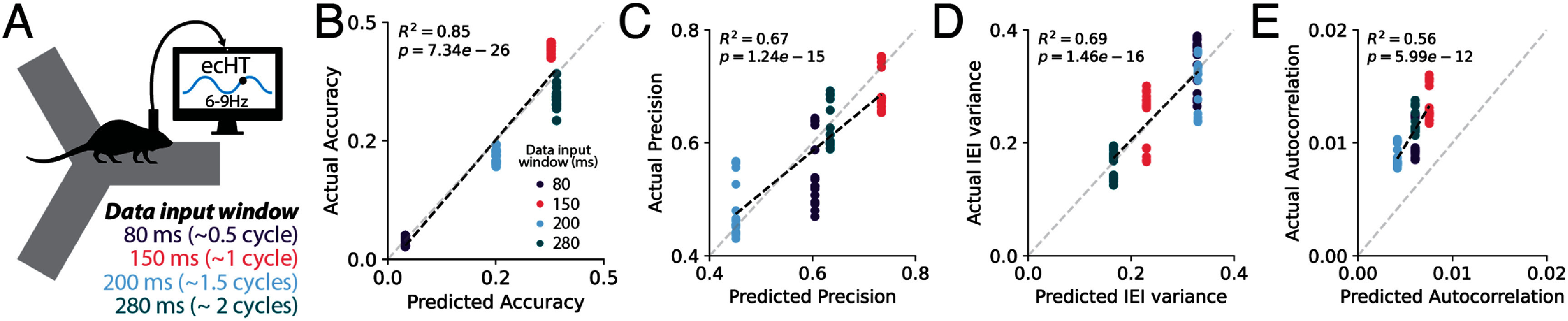
*In vivo* validation of ecHT algorithm performance with four different input data window lengths. (A) Schematic of *in vivo* real-time experiment. Two rats were implanted with recording electrodes in the dorsal CA1 region of the hippocampus. ecHT was used to detect peaks (*π*) in theta oscillations (6–9 Hz) in real time as animals ran on a Y maze. We tested various input data window lengths (colored text). (B) High correlation between observed versus predicted accuracy for four different input data window lengthes (80 ms: purple, 150 ms: pink, 200 ms: blue, 280 ms: green). Each point represents results from one 10 min session. Linear regression (*R*^2^ = 0.95, *p* = 9.97 × 10^−41^). (C) High correlation between observed versus predicted precision for four different input data window lengths. Linear regression (*R*^2^ = 0.67, *p* = 1.25 × 10^−15^). (D) High correlation between observed versus predicted inter-event interval for four different input data window lengths. Linear regression (*R*^2^ = 0.69, *p* = 1.46 × 10^−16^). (E) High correlation between observed versus predicted autocorrelation for four different input data window lengths. Linear regression (*R*^2^ = 0.56, *p* = 5.99 × 10^−12^).

Our *in silico* evaluations showed that Fourier-based phase detection algorithms can be optimized by matching the input data window length with the period of the oscillation (figure 4). We performed an *in vivo* real-time experiment to validate this prediction (figure 8(A)). We selected the ecHT algorithm since it performed consistently better compared with HT or PM. We selected four window lengths, 80 ms, 150 ms, 200 ms and 280 ms, corresponding to 0.5, 1, 1.5 and 2 cycles of the theta oscillation respectively. Based on our simulation data (figure 4 Rodent LFP), these window lengths should produce distinct performance results. We applied ecHT to detect the peak of the theta oscillation in the dorsal CA1 region of the hippocampus in two rats as they navigated a Y maze for reward. The observed real-time performance was highly correlated with our predictions (accuracy: *R*^2^ = 0.95, *p* = 9.97 × 10^−41^; precision: *R*^2^ = 0.67, *p* = 1.24 × 10^−15^; IEI variance: *R*^2^ = 0.69, *p* = 1.46 × 10^−16^; autocorrelation: *R*^2^ = 0.56, *p* = 5.99 × 10^−12^). The best performance across metrics was observed for an input data window corresponding to approximately one cycle of the oscillation, followed by two cycles. The other window lengths, 0.5 and 1.5 cycles, performed less well. For accuracy, an input data window size of 1.5 cycles reduced the performance by half compared with a window length of one cycle (figure 8(B)), pointing to the importance of matching the input data window length to the period of the oscillation.

## 3. Discussion

Closed-loop phase-specific neurostimulation is a powerful method to modulate ongoing brain activity for clinical applications and basic research (Widge and Miller 2019, Wendt *et al* 2022, Widge 2024). For clinical applications, phase-specific stimulation has been used to terminate epileptic seizures (Takeuchi *et al* 2021), suppress essential tremor (Brittain *et al* 2013, Cagnan *et al* 2013, Schreglmann *et al* 2021), augment sedation (Smith *et al* 2024) and modulate sleep patterns (Bressler *et al* 2023, 2024, Dong *et al* 2023). In basic neuroscience research, phase-locked stimulation has been used to modulate corticospinal excitability and synaptic plasticity (Zanos *et al* 2018, Wischnewski *et al* 2022, Zrenner *et al* 2023), investigate phase-specific function of neural oscillations in cognition (Ngo *et al* 2013, Siegle and Wilson 2014, Kanta *et al* 2019, Knudsen and Wallis 2020, Mankin and Fried 2020, Lurie *et al* 2022, Xie *et al* 2023). Optimizing the performance of online phase detection algorithms and closed-loop phase-specific stimulation systems will benefit these applications.

Our work identifies one method for optimizing the performance of Fourier transform-based algorithms that is convenient to implement. We found that performance can be optimized by adjusting the input data window to match the duration of one cycle of the oscillation. This was shown with *in silico* evaluations and validated with an *in vivo* real-time experiment in rats. Other parameters, such as the filter type and the filter order, have a less significant impact. Our evaluation focuses on the theta oscillation in rodents, the alpha oscillation and essential tremor kinematics in humans; whether our method can generalize to higher frequency oscillations is a topic for future investigation. Higher frequency oscillations such as beta (12–30 Hz) and gamma (30–90 Hz) oscillations may have different spectral properties and may require further optimization. One implication of our study is that users may need to balance the trade-off between accuracy and temporal consistency when deciding on the input data window length for their application. This decision could depend on the biological signal targeted in each application. If the consistency of the output sequence across cycles is more important than the accuracy of individual outputs, then increasing the input data window length could be useful. This can be evaluated using methods we have shown here, by recording the data and performing *in silico* analysis to determine the optimal input data window length for each application. Overall, our work suggests starting the optimization process with an input data window length corresponding to approximately one cycle for the oscillation of interest.

Whether different algorithm classes are sensitive to input data window length remains to be determined. We found that the PM algorithm is less sensitive to window length variations as it does not involve time-frequency transformation. Phase estimation algorithms using machine learning and state space modeling approaches also do not involve time-frequency transformation, and thus may rely less on a well-chosen window length (Mcintosh and Sajda 2020, Rosenblum *et al* 2021, Wodeyar *et al* 2021, Busch *et al* 2022, Tseng *et al* 2023). Additional classes of phase estimation algorithms include autoregressive forecast to address the Gibbs phenomenon seen in online filtering (Chen *et al* 2013, Blackwood *et al* 2018, Onojima and Kitajo 2021, Schatza *et al* 2022), Kalman filtering to adjust frequency filter range in real time or Genetic algorithms to search for a set of globally optimal parameters (Van Zaen *et al* 2010, Chen *et al* 2013, Rodriguez Rivero and Ditterich 2021).

In addition to phase-specific stimulation, many closed-loop laboratory experiments involve a non-phase-specific condition as control. The objective can be to ensure that the effects observed with phase-specific stimulation are unique to the phase of the stimulation (Cagnan *et al* 2017, Knudsen and Wallis 2020, Lurie *et al* 2022, Chen *et al* 2024). This objective requires a similar number of stimulations to be delivered but without phase preference. In other studies, the objective is instead to eliminate the rhythmicity of the underlying neural activity by delivering an arrhythmic stimulation sequence (Kanta *et al* 2019, Schreglmann *et al* 2021). Non-phase-specific stimulation approaches include open-loop stimulation at fixed time intervals (Ngo *et al* 2013, Schreglmann *et al* 2021, West *et al* 2022, Xie *et al* 2023), stimulation at randomly determined time points (Hampson *et al* 2018, Kanta *et al* 2019, Knudsen and Wallis 2020, Roeder *et al* 2024) and replaying phase-specific stimulation sequence at a later time in the same brain region (Zanos *et al* 2018, West *et al* 2022). Although closed-loop random phase generation approach produces greater phase randomness than the open loop approach, the open loop approach produces more temporally randomized outputs. These results indicate that different random-phase-generating algorithms vary in the randomness of their output, and the choice of the control algorithm needs to be tailored to the experiment.

The choice of random-phase-generating algorithm for an experiment depends on the application requirements and the signal type, as we have shown in figure 6. For example, it may not be practical in some cases to predefine the experiment duration to assign random phases, which excludes the random schedule algorithm. Additionally, some rhythmic biological processes may be more robust to perturbation. Residual rhythmicity resulting from a slightly less random neural stimulation algorithm could be sufficient to retain function, resulting in the lack of biological changes. In this case, priority should be given to eliminating rhythmicity, which favors random delay or random schedule algorithms. Otherwise, if the desired output from an algorithm is random, then the random target algorithm would be most effective.

We found frequency-specific SNR, and amplitude and frequency variability to be important factors in algorithm performance. Several definitions of SNR exist that describe the relative strength of the rhythm of interest versus other spectral components (Donoghue *et al* 2020, 2022, Shirinpour *et al* 2020, Zrenner *et al* 2020, Tseng *et al* 2023) These features of the signal have been shown to affect the performance of the algorithm (Shirinpour *et al* 2020, Wodeyar *et al* 2021, Tseng *et al* 2023). We also identified frequency and amplitude variability as two spectral properties of the input signals that impair algorithm performance. We found that the phase estimation error is positively correlated with frequency variability within the input data window. Thus, oscillations with high frequency variability may pose a challenge for phase estimation using FFT-based methods. We indeed found that amplitude and frequency variations are negatively correlated with algorithm performance. Our finding that a one-cycle input data window works best for FFT-based methods can also be explained by frequency and amplitude variability. If the input data window contains more than one cycle of signal, the signal within the window carries more frequency and amplitude variations, which increases phase estimation error and worsens algorithm performance. Our study shows that optimal performance for an algorithm depends on the properties of the input signal; algorithm parameters, such as input data window length, may need to be tuned to the specific signal of interest. Future designs of phase detection algorithms may benefit from explicitly calculating signal properties and using the information for real-time adaptation (Van zaen *et al* 2010, Chen *et al* 2013, Rodriguez rivero and Ditterich 2021).

The phase detection algorithms evaluated here perform best with biological signals with stable amplitude and frequency in the frequency band of interest, such as human essential tremor signals. This suggests that signals with burst-like properties, where sudden amplitude changes can be large, may require additional methods to optimize performance. Another potential challenge for Fourier-based methods is mixed-frequency signals. However, a single type of rhythmic biological signal usually spans a well-defined, continuous frequency range. Thus, a bandpass filter is typically used to isolate the rhythmic signal of interest. If a hypothetical signal of interest has mixed-frequency components that span several disjoint frequency ranges, one phase estimation algorithm may be needed for each contiguous frequency band of interest. A logical operation can then be used to determine how the final output decision is calculated from each algorithm. We also found some algorithms and signal combinations produced outputs that were biased relative to the target (Supp. figure 10, Supp. table 6). One method to counter the bias is to shift the detection target to a value earlier or later than the desired phase, to offset the general bias. The amount of target shift should match the magnitude of the bias, and the direction of target shift should be the opposite of the bias direction. This may involve empirical testing or *in silico* predictions to determine the best shifted target to offset the bias of a specific signal and algorithm combination. We also examined the effect of transient, large-amplitude artifacts caused by equipment disconnection and movement. These are different from the aperiodic background activity quantified by frequency-specific SNR. As expected, these recording artifacts decreased performance across all metrics (Supp. figure 9). Managing these artifacts in real time requires an additional step, typically an amplitude threshold mechanism that detects the onset of large-amplitude artifacts and temporally gates the output of the phase estimation algorithm until signal amplitude returns to baseline levels. As mentioned previously, the Fourier-based methods perform best on signals that have stable amplitude and frequency properties.

Lastly, other classes of phase detection algorithms may offer advantages over the Fourier-based ones. Fourier-based methods are attractive because they are more convenient to implement and deploy, analytically tractable, and well validated. They tend to work well for oscillations with one dominant frequency that is relatively stable. However, some biological signals vary in amplitude and frequency on a fast timescale in the form of burst-like events, posing challenges for Fourier-based phase estimation. These challenges could be overcome with newer machine learning or state space algorithms, which can potentially perform better on non-stationary biological signals (Mcintosh and sajda 2020, Rosenblum *et al* 2021, Wodeyar *et al* 2021, Busch *et al* 2022, Tseng *et al* 2023). These approaches often require training data, parameter tuning, or more complex infrastructure for real-time deployment. Another advantage offered by these non-Fourier-based algorithms is their ability to manage real-time noise and artifacts. More sophisticated models could incorporate artifact detection and isolation to reduce the impact of real-time artifacts in phase estimation. These requirements are especially important for mission-critical real-time applications.

## 4. Methods

### 4.1. Data

We chose real-world biological signals from a human EEG dataset (Unsworth *et al* 2014), an essential tremor hand kinematics dataset (Schreglmann *et al* 2021) and a rodent LFP dataset collected in our lab. Rodent LFP data were recorded from the pyramidal layer of the dorsal hippocampus CA1 region of rats (*N* = 11, male, 44 sessions, 10 min each) performing spatial navigation tasks. The data was recorded at a sampling rate of 1500 Hz. Human EEG data (*N* = 40, 40 sessions, 50 min each) was recorded at 150 Hz from the POZ electrode over the posterior brain of human subjects performing a working memory task. Essential tremor kinematics data (*N* = 11, 346 sessions, 1 min each) were recorded at 250 Hz from an accelerometer attached to the finger of tremor patients receiving non-invasive tremor suppression therapy.

### 4.2. Rodent LFP data collection

All procedures were performed under approval by the University’s Institutional Animal Care and Use Committee, according to the guidelines of the Association for Assessment and Accreditation of Laboratory Animal Care. Long Evans rats (*N* = 11, Charles River Laboratories) had adjustable tetrodes targeting the dorsal CA1 region of the hippocampus (AP −3.8, ML −2.75 relative to Bregma). Tetrodes were adjusted into the dorsal CA1 region over two to three weeks after surgical implantation. Animals navigated elevated mazes (Y or F shaped) for food rewards for 10 min (up to 4 session per day, up to 5 days). Data were recorded at 1500 Hz using a 128-channel digitizing headstage and the Trodes data recording interface (SpikeGadgets LLC).

### 4.3. Reference phase calculation

We define the ‘reference phase’ as the phase of the signal calculated offline (Zrenner *et al* 2020). We first filtered the raw signal using a non-causal second order Butterworth filter for the frequency band of interest. We then used the HT to calculate the analytic signal from the filtered signal. We then derived instantaneous phases from the analytic signal. This was done using the SciPy package in Python (signal.butter, signal.sosfiltfilt and signal.Hilbert).

### 4.4. Signal property quantification

We measured the following spectral properties in each recording: frequency-specific SNR, amplitude variability and frequency variability. We calculated the SNR in the frequency range of interest as the ratio between the log observed power and the estimated aperiodic background at the spectral peak’s central frequency (Donoghue *et al* 2022, Wilson *et al* 2022). This was done using the Fitting Oscillations & One-Over-F (FOOOF) method (fooof-tools.github.io) (Donoghue *et al* 2020). Specifically, we used the welch function from the SciPy library to calculate power spectral density then used the FOOOF library to estimate the aperiodic component. We selected the power peak best aligned with our frequency range of interest and identified the central frequency of the peak. We calculated SNR (dB) as the difference between the log observed power (dB) and aperiodic power (dB) of the power peak.

We measured signal amplitude variability using nPVI. We extracted the instantaneous amplitude envelope for the frequency range of interest. We calculated the mean amplitude for each cycle and calculated the average amplitude difference between consecutive cycles as follows:

\begin{align*}{\text{nPVI}} = \frac{1}{{N - 1}}\mathop \sum \limits_{k = 1}^{N - 1} \left| {\frac{{{d_k} - {d_{k + 1}}}}{{\left( {{d_k} + {d_{k + 1}}} \right)/2}}} \right|\end{align*}

where *N* is the total number of cycles in the oscillation, and *d_k_* is the average amplitude in cycle *k*.

We calculated frequency variability as the variance of the instantaneous frequency across time. We filtered the signal into the frequency range of interest and then used the HT to extract the instantaneous frequency. The variance of instantaneous frequency across time was then calculated and normalized by the width of the frequency range of interest. We calculated two types of frequency variability: ‘mean window variability’, which is calculated as the variance of the instantaneous frequency within each data window fed into the algorithm, and then averaged across all windows, and ‘total frequency variability’, which is the variance of the instantaneous frequency for the entire recording session. The fraction of overall frequency variability (figure S5 (A)–(C)) is computed as the ‘mean window variability’ divided by the ‘total frequency variability’. We found that longer data windows show higher mean window variability, and this variability approaches the total signal variability as window length increases.

### 4.5. Phase detection algorithms

We evaluated the performance of three phase detection algorithms by running them on the biological signals described above. To emulate real-time operations, we iterated stepwise across the signal to extract data from a window corresponding to the last *N* samples (input data window). Data from this window was then processed by the phase detection algorithm to produce an instantaneous phase estimate for the last sample in the data window (endpoint phase).

The ecHT (Schreglmann *et al* 2021) reduces the effect of the Gibbs phenomenon when estimating the endpoint phase of a signal. It performs a FFT on the raw signal in the input data window. The negative frequency components of the Fourier spectrum are eliminated, and the positive frequency components are multiplied by two. Frequency components outside the frequency range of interest are then eliminated, and the processed frequency components are transformed into the analytic signal using an inverse Fourier transform. The instantaneous phase at the last sample of the analytic signal is used as the phase estimate.

The HT algorithm first bandpass-filters the input data to the desired frequency range. It then computes the analytic signal of the filtered signal using a HT and uses the instantaneous phase at the last sample as the phase estimate.

The PM algorithm first bandpass-filters the input data to the desired frequency range then performs linear regression on the bandpass-filtered signal. It detects changes in the sign of the slope to identify a peak or trough, corresponding to a half-cycle of the oscillation. PM assumes a linear relationship between phase and the number of samples elapsed (linear phase progression). It calculates the change in phase per signal sample using the duration of the half-cycle in number of samples. The ratio is used to extrapolate the instantaneous phase for future samples, until the next half-cycle is reached.

For all three phase detection algorithms, we chose a specific target phase and issued an output when the estimated instantaneous phase reached the target phase. To avoid repeated outputs within the same cycle, the output was then disabled until the estimated start of the next cycle.

### 4.6. Algorithm output evaluation

Phase detection algorithms produced output when the estimated instantaneous phase reached a predefined phase target. The output events are timestamps when the algorithm issued outputs. We used the following metrics to quantify the quality of the algorithm’s output.

### 4.7. Accuracy

Accuracy is defined as the proportion of output events with phases within a *π*/4 tolerance range centered around the target phase,

\begin{align*}{\text{accuracy = }}\frac{{{\text{number of accurate stimulation events}}}}{{{\text{total number of stimulation events}}}}.\end{align*}

We set the tolerance range to be *π*/4 wide (45° or one eighth of a cycle) because this is likely to provide sufficient coverage of the cycle based on known biological functions associated with the cycle. For example, in rodents, distinct biological processes have been associated with one half of a theta oscillation cycle (*π* or 180°) (Wang *et al* 2020, Robinson and Brandon 2021). Neurons usually have spiking that spans half a theta cycle. We reasoned that an accuracy that is half of the biologically relevant timescale should be sufficiently accurate to evaluate the performance of algorithms designed for phase detection.

### 4.8. Precision

Precision reports the consistency of the algorithm output. The resultant vector length is an ideal metric for evaluating consistency. The resultant vector is computed as follows:

\begin{align*}\overline{\alpha} = \frac{1}{N}\left\|\mathop \sum \limits_{j = 1}^N {\text{exp}}\left( {i \cdot {\alpha _j}} \right)\right\| \end{align*}

where *N* is the number of output events, ${\alpha _j}$ are the individual detected phases and $\overline{\alpha} $ is the resultant phase vector. The resultant vector length is the magnitude of the resultant phase vector $\overline{\alpha} $. If an algorithm produces an output at the desired phase on every cycle of an oscillation, all output events will have identical phases, and the resultant vector length will be one. If the algorithm produces a different phase output on every cycle, then the resultant vector length will be zero. The resultant vector length can thus capture how consistent the algorithm outputs are and can be used as a measure of precision.

### 4.9. IEI variance

The IEI between two consecutive output events is defined as the ground truth phase distance between the two events. Variance of the IEI distribution is then calculated as follows:

\begin{align*}{\sigma ^2} = \frac{1}{{N - 1}}\mathop \sum \limits_{j = 1}^{N - 1} \left( {{\text{IE}}{{\text{I}}_j} - \overline {{\text{IEI}}} } \right)\end{align*}

where *N* is the number of output events, ${\text{IE}}{{\text{I}}_j}$ is the IEI between the *j*th and the *j* + *1*th event and $\overline {{\text{IEI}}} $ is the mean IEI of the output sequence. The IEI variance is ideally zero if all events are generated at the target phase on each cycle. However, the performance of the algorithms on biological signals is not perfect, and the algorithm outputs are not at the target phase on every cycle. Therefore, IEI values can range from 0 to 4*π* if the algorithm outputs every cycle, or arbitrarily large if the algorithm fails to detect the target phase in multiple cycles.

### 4.10. Autocorrelation

This metric measures the rhythmic regularity of the output sequence in phase. We calculated the autocorrelation (80-radian windows) for the sequence of unwrapped phase events. The autocorrelation was normalized to produce a probability density function. We then averaged the autocorrelation values at integer multiples of 2*π*. If an algorithm correctly produces a single output at the target phase on every cycle, there would be regular peaks in the autocorrelation at intervals of 2*π*. On the other hand, if detections are irregular, the autocorrelation will have weaker periodic peaks. This metric provides a measure of how regularly the algorithm outputs in phase across cycles, with higher autocorrelation at 2*π*−spaced lags indicating more rhythmically consistent output.

### 4.11. Algorithm optimization

The phase detection algorithms have three tunable parameters: the type of bandpass filter, the order of the bandpass filter and the input data window length. We optimized these parameters for each algorithm using a grid search. The optimization space included parameter combinations across the three types of filters, filter orders from 2 to 6 and input data window lengths ranging from 5 ms to 500 ms in steps of 5 ms. We included the Butterworth, Chebyshev type I and Elliptic filters. We started with filter order 2 to improve isolation for signals in the frequency band of interest. For each parameter combination, we ran the algorithm on recorded data from all three signal types to detect the peak in each cycle as the target. We then calculated the accuracy, precision, IEI variance and autocorrelation for the output from each parameter combination. This was done using 20 recordings from each signal type.

### 4.12. Random-phase-generating algorithms

We also evaluated algorithms that produce output events at random phases, which we refer to as random-phase-generating algorithms. The “random target” algorithm uses the ecHT algorithm to estimate instantaneous phase but selects the output phase target on each cycle by randomly selecting a value from a uniform von Mises distribution (*μ* = *π, κ* = 0). The “random delay” algorithm is also based on ecHT but instead produces an output after a random time delay, between 0 and the period of the oscillation, relative to the start of each cycle. The period of the cycle is determined based on the central frequency for the oscillation of interest. The “random schedule” algorithm involves predefining the timing of the output events relative to the start of the experiment based on the estimated experiment duration and the desired number of output events. This algorithm is blind to the ongoing oscillation and does not perform phase estimation.

### 4.13. Relating signal property to algorithm performance variations

To quantify the importance of signal properties (SNR, amplitude variability and frequency variability) to algorithm performance, we performed linear regression between each signal property and each algorithm performance metric (SciPy, Scikit-learn). For each regression, we used the signal property and the algorithm performance data from all sessions for each signal type.

### 4.14. *In vivo* testing

Two Long Evans rats (male, 45 and 59 weeks old) were implanted with tetrodes targeting the dorsal CA1 region of the hippocampus. We collected data as the animals navigated a Y-shaped maze for reward (15 sessions, 10 minutes each). Data was streamed at 1500 Hz using a 128-channel digitizing headstage and the Trodes data recording interface (SpikeGadgets LLC). For real-time signal processing, LFP data from a single channel was streamed to our Python-based ecHT processor. The function detects the peak of the theta oscillation. We concurrently ran four separate, independent instances of the phase detection function, with each instance using a different input data window length. This ensured that the same signal was used to test the effects of varying data input window length in real time.

### 4.15. Statistical tests

We used non-parametric tests for between-group comparisons where the data in each group followed a continuous distribution. To determine whether data from two or more groups came from the same distribution, we used the Kruskal–Wallis test. This was used for between-group statistical testing of the distributions of signal features (SNR, amplitude variability and frequency variability). To determine whether the median of a distribution was centered at 0, we used the one-sample Wilcoxon signed-rank test. To test whether distributions had similar variance, we used the Levene’s test. For comparisons of algorithm performance between algorithms, signal types and detection targets, we used three-way, non-parametric permutation-based ANOVA.

## Data Availability

The data cannot be made publicly available upon publication because the cost of preparing, depositing and hosting the data would be prohibitive within the terms of this research project. The data that support the findings of this study are available upon reasonable request from the authors. Supplementary data 1 available at https://doi.org/10.1088/1741-2552/ae10e1/data1.

## References

[jneae10e1bib1] Blackwood E, Lo M C, Alik Widge S (2018). Continuous phase estimation for phase-locked neural stimulation using an autoregressive model for signal prediction. 2018 40th Annual Int. Conf. IEEE Engineering in Medicine and Biology Society.

[jneae10e1bib2] Bressler S, Neely R, Yost R M, Wang D (2024). A randomized controlled trial of alpha phase-locked auditory stimulation to treat symptoms of sleep onset insomnia. Sci. Rep..

[jneae10e1bib3] Bressler S, Neely R, Yost R M, Wang D, Read H L (2023). A wearable EEG system for closed-loop neuromodulation of sleep-related oscillations. J. Neural Eng..

[jneae10e1bib4] Brittain J S, Probert-smith P, Aziz T Z, Brown P (2013). Tremor suppression by rhythmic transcranial current stimulation. Curr. Biol..

[jneae10e1bib5] Busch J L, Feldmann L K, Kühn A A, Rosenblum M (2022). Real-time phase and amplitude estimation of neurophysiological signals exploiting a non-resonant oscillator. Exp. Neurol..

[jneae10e1bib6] Buzsáki G (2002). Theta oscillations in the hippocampus. Neuron.

[jneae10e1bib7] Buzsáki G, Moser E I (2013). Memory, navigation and theta rhythm in the hippocampal-entorhinal system. Nat. Neurosci..

[jneae10e1bib8] Cagnan H (2013). Phase dependent modulation of tremor amplitude in essential tremor through thalamic stimulation. Brain.

[jneae10e1bib9] Cagnan H (2017). Stimulating at the right time: phase-specific deep brain stimulation. Brain.

[jneae10e1bib10] Chen C (2024). The dynamic state of a prefrontal-hypothalamic-midbrain circuit commands behavioral transitions. Nat. Neurosci..

[jneae10e1bib11] Chen L L, Madhavan R, Rapoport B I, Anderson W S (2013). Real-time brain oscillation detection and phase-locked stimulation using autoregressive spectral estimation and time-series forward prediction. IEEE Trans. Biomed. Eng..

[jneae10e1bib12] Dong S, Xie Z, Yuan Y (2023). Transcranial ultrasound stimulation modulates neural activities during NREM and REM depending on the stimulation phase of slow oscillations and theta waves in the hippocampus. Cereb. Cortex.

[jneae10e1bib13] Donoghue T (2020). Parameterizing neural power spectra into periodic and aperiodic components. Nat. Neurosci..

[jneae10e1bib14] Donoghue T, Schaworonkow N, Voytek B (2022). Methodological considerations for studying neural oscillations. Eur. J. Neurosci..

[jneae10e1bib15] Halgren M (2019). The generation and propagation of the human alpha rhythm. Proc. Natl Acad. Sci. USA.

[jneae10e1bib16] Hampson R E (2018). Developing a hippocampal neural prosthetic to facilitate human memory encoding and recall. J. Neural Eng..

[jneae10e1bib17] Hyman J M, Wyble B P, Goyal V, Rossi C A, Hasselmo M E (2003). Stimulation in hippocampal region CA1 in behaving rats yields long-term potentiation when delivered to the peak of theta and long-term depression when delivered to the trough. J. Neurosci..

[jneae10e1bib18] Kanta V, Pare D, Headley D B (2019). Closed-loop control of gamma oscillations in the amygdala demonstrates their role in spatial memory consolidation. Nat. Commun..

[jneae10e1bib19] Kim B, Erickson B A, Fernandez-nunez G, Rich R, Mentzelopoulos G, Vitale F, Medaglia J D (2023). EEG phase can be predicted with similar accuracy across cognitive states after accounting for power and signal-to-noise ratio. eNeuro.

[jneae10e1bib20] Klimesch W, Sauseng P, Gerloff C (2003). Enhancing cognitive performance with repetitive transcranial magnetic stimulation at human individual alpha frequency. Eur. J. Neurosci..

[jneae10e1bib21] Knudsen E B, Wallis J D (2020). Closed-loop theta stimulation in the orbitofrontal cortex prevents reward-based learning. Neuron.

[jneae10e1bib22] Louis E D, Faust P L (2020). Essential tremor pathology: neurodegeneration and reorganization of neuronal connections. Nat. Rev. Neurol..

[jneae10e1bib23] Lurie S M, Kragel J E, Schuele S U, Voss J L (2022). Human hippocampal responses to network intracranial stimulation vary with theta phase. Elife.

[jneae10e1bib24] Mankin E A, Fried I (2020). Modulation of human memory by deep brain stimulation of the entorhinal-hippocampal circuitry. Neuron.

[jneae10e1bib25] Mansouri F, Dunlop K, Giacobbe P, Downar J, Zariffa J (2017). A fast EEG forecasting algorithm for phase-locked transcranial electrical stimulation of the human brain. Front. Neurosci..

[jneae10e1bib26] Mcintosh J R, Sajda P (2020). Estimation of phase in EEG rhythms for real-time applications. J. Neural Eng..

[jneae10e1bib27] Ngo H V, martinetz T, Born J, Mölle M (2013). Auditory closed-loop stimulation of the sleep slow oscillation enhances memory. Neuron.

[jneae10e1bib28] Onojima T, Kitajo K (2021). A state-informed stimulation approach with real-time estimation of the instantaneous phase of neural oscillations by a Kalman filter. J. Neural Eng..

[jneae10e1bib29] Pantazatos S P (2023). The timing of transcranial magnetic stimulation relative to the phase of prefrontal alpha EEG modulates downstream target engagement. Brain Stimul..

[jneae10e1bib30] Peylo C, hilla Y, Sauseng P (2021). Cause or consequence? Alpha oscillations in visuospatial attention. Trends Neurosci..

[jneae10e1bib31] Robinson J C, Brandon M P (2021). Skipping ahead: a circuit for representing the past, present, and future. Elife.

[jneae10e1bib32] Rodriguez Rivero C, Ditterich J (2021). A user-friendly algorithm for adaptive closed-loop phase-locked stimulation. J. Neurosci. Methods.

[jneae10e1bib33] Roeder B M (2024). Developing a hippocampal neural prosthetic to facilitate human memory encoding and recall of stimulus features and categories. Front. Comput. Neurosci..

[jneae10e1bib34] Rosenblum M, Pikovsky A, Kühn A A, Busch J L (2021). Real-time estimation of phase and amplitude with application to neural data. Sci. Rep..

[jneae10e1bib35] Schatza M J, Blackwood E B, Nagrale S S, Widge A S (2022). Toolkit for oscillatory real-time tracking and estimation (TORTE). J. Neurosci. Methods.

[jneae10e1bib36] Schreglmann S R (2021). Non-invasive suppression of essential tremor via phase-locked disruption of its temporal coherence. Nat. Commun..

[jneae10e1bib37] Shirinpour S, Alekseichuk I, Mantell K, Opitz A (2020). Experimental evaluation of methods for real-time EEG phase-specific transcranial magnetic stimulation. J. Neural Eng..

[jneae10e1bib38] Siegle J H, Wilson M A (2014). Enhancement of encoding and retrieval functions through theta phase-specific manipulation of hippocampus. Elife.

[jneae10e1bib39] Smith S K, kafashan M, Rios R L, Brown E N, Landsness E C, Guay C S, Palanca B J A (2024). Daytime dexmedetomidine sedation with closed-loop acoustic stimulation alters slow wave sleep homeostasis in healthy adults. BJA Open.

[jneae10e1bib40] Takeuchi Y, Harangozó M, Pedraza L, Földi T, Kozák G, Li Q, Berényi A (2021). Closed-loop stimulation of the medial septum terminates epileptic seizures. Brain.

[jneae10e1bib41] Tseng C H, Chen J H, Hsu S M (2023). The effect of the peristimulus α phase on visual perception through real-time phase-locked stimulus presentation. eNeuro.

[jneae10e1bib42] Unsworth N, Fukuda K, Awh E, Vogel E K (2014). Working memory and fluid intelligence: capacity, attention control, and secondary memory retrieval. Cogn. Psychol..

[jneae10e1bib43] Van Zaen J, Uldry L, Duchêne C, Prudat Y, Meuli R A, Murray M M, Vesin J M (2010). Adaptive tracking of EEG oscillations. J. Neurosci. Methods.

[jneae10e1bib44] Wang M, Foster D J, Pfeiffer B E (2020). Alternating sequences of future and past behavior encoded within hippocampal theta oscillations. Science.

[jneae10e1bib45] Welton T, Cardoso F, Carr J A, Chan L L, Deuschl G, Jankovic J, Tan E K (2021). Essential tremor. Nat. Rev. Dis. Primers.

[jneae10e1bib46] Wendt K, Denison T, Foster G, Krinke L, Thomson A, Wilson S, Widge A S (2022). Physiologically informed neuromodulation. J. Neurol. Sci..

[jneae10e1bib47] West T O, Magill P J, Sharott A, Litvak V, Farmer S F, Cagnan H (2022). Stimulating at the right time to recover network states in a model of the cortico-basal ganglia-thalamic circuit. PLoS Comput. Biol..

[jneae10e1bib48] Widge A S (2024). Closing the loop in psychiatric deep brain stimulation: physiology, psychometrics, and plasticity. Neuropsychopharmacology.

[jneae10e1bib49] Widge A S, Miller E K (2019). Targeting cognition and networks through neural oscillations: next-generation clinical brain stimulation. JAMA Psychiatry.

[jneae10e1bib50] Wilson L E, da Silva Castanheira J, Baillet S (2022). Time-resolved parameterization of aperiodic and periodic brain activity. Elife.

[jneae10e1bib51] Wischnewski M, Haigh Z J, Shirinpour S, Alekseichuk I, Opitz A (2022). The phase of sensorimotor mu and beta oscillations has the opposite effect on corticospinal excitability. Brain Stimul..

[jneae10e1bib52] Wodeyar A, Schatza M, Widge A S, eden U T, Kramer M A (2021). A state space modeling approach to real-time phase estimation. Elife.

[jneae10e1bib53] Xie Z, Dong S, Zhang Y, Yuan Y (2023). Transcranial ultrasound stimulation at the peak-phase of theta-cycles in the hippocampus improve memory performance. Neuroimage.

[jneae10e1bib54] Zanos S, Rembado I, Chen D, Fetz E E (2018). Phase-locked stimulation during cortical beta oscillations produces bidirectional synaptic plasticity in awake monkeys. Curr. Biol..

[jneae10e1bib55] Zrenner C, Galevska D, Nieminen J O, Baur D, Stefanou M I, Ziemann U (2020). The shaky ground truth of real-time phase estimation. Neuroimage.

[jneae10e1bib56] Zrenner C, Kozák G, Schaworonkow N, Metsomaa J, BAUR D, Vetter D, Blumberger D M, Ziemann U, Belardinelli P (2023). Corticospinal excitability is highest at the early rising phase of sensorimotor *µ*-rhythm. Neuroimage.

